# Homage to Chris Dobson

**DOI:** 10.3389/fmolb.2019.00137

**Published:** 2019-12-12

**Authors:** Jean Baum, Fabrizio Chiti, Alfonso De Simone, Tuomas P. J. Knowles, Janet R. Kumita, Sheena E. Radford, Carol V. Robinson, Xavier Salvatella, Karen Valelli, Michele Vendruscolo, Annalisa Pastore, Gian Gaetano Tartaglia

**Affiliations:** ^1^School of Arts and Sciences, Rutgers, The State University of New Jersey, New Brunswick, NJ, United States; ^2^Department of Experimental and Clinical Biomedical Sciences, University of Florence, Florence, Italy; ^3^Department of Life Sciences, Imperial College London, London, United Kingdom; ^4^Centre for Misfolding Diseases, Department of Chemistry, University of Cambridge, Cambridge, United Kingdom; ^5^Faculty of Biological Sciences, Astbury Centre for Structural Molecular Biology, School of Molecular and Cellular Biology, University of Leeds, Leeds, United Kingdom; ^6^Physical and Theoretical Chemistry Laboratory, University of Oxford, Oxford, United Kingdom; ^7^ICREA, Institute for Research in Biomedicine and the Barcelona Institute of Science and Technology, Barcelona, Spain; ^8^Maurice Wohl Institute & Dementia Research Institute, King's College London, London, United Kingdom; ^9^Centre for Genomic Regulation (CRG), Barcelona Institute of Science and Technology (BIST), Catalan Institute for Research and Advance Studies (ICREA), Barcelona, Spain

**Keywords:** protein chemistry, NMR, protein folding, amyloid, protein aggregation

**Graphical Abstract:**
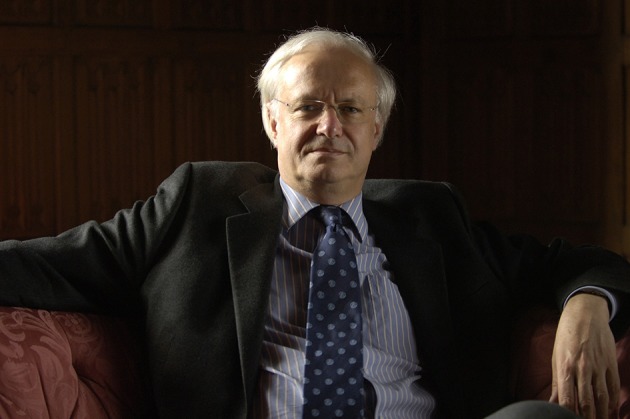
A recent picture of Chris Dobson in his room in college.

**Sir Chris Dobson** (1949-2019) has been an inspiring model of a scientist for all of us. Open-minded, curious, and collaborative, he touched our lives by idiosyncratically understanding both scientific and personal aspects of science. He could motivate people by providing unique insights in experimental and theoretical matters. In his busy days as Master of St. John's, he could always find the time to listen to people and discuss things.

With the purpose of remembering Chris, we collected messages from present and former collaborators. The image of Chris that comes out in this way is more choral and vibrant than what could come from more thorough but more formal obituaries. We apologize for the many colleagues who were not included because of time and space constraints.

**Jean Baum (Professor, Rutgers School of Art and Sciences, US, in Chris' lab at Oxford in January 1996-August 1998 and in Cambridge for a sabbatical in Spring 2019)**. I was considering Chris' lab in Oxford for a postdoc in 1986 and wrote to him and asked if I could meet him at a Biophysical Society meeting that he was attending. I was graduating from Alex Pines' lab and, for my postdoc, was considering a transition from solid-state NMR to biological solution NMR. At this first meeting, Chris' description of his work was inspiring. He presented the big questions in protein folding, he made a few Chris-like jokes, and I decided to join his lab.

The biggest lesson that Chris taught me was to be open-minded, collaborative, and scientifically curious. When I was in his lab everything was interesting to him—from small changes in chemical shifts to why alpha-lactalbumin would form a gel at low pH. Chris had a tremendous ability to tell a story and to think outside the box and he worked hard to instill this with his students. On the personal side, Chris was a role model for embracing life, for “work hard/play hard.”

Chris touched so many lives—students, postdocs, colleagues, friends, spouses, and more. He opened up new, critically important, scientific vistas and trained so many students. We all have “Chris stories” and I would like to share the following: I was speaking at a Biopolymers Gordon conference in the early 1990's and I brought my 3-year old boy to the meeting. At the time, the Gordon Conference rules were that my son and I needed to be housed in a separate dorm from everyone else and that we needed to eat on the other side of the dining hall from my scientific colleagues in order to not disturb them. Chris was extremely unhappy about this and on the second night made a bold statement by coming over to my side of the dining hall, picking up my son and asking Mark to sit in his lap while we all ate dinner together on the “science” side of the dining room. We continued with this “non-allowed” seating arrangement for the rest of the meeting. Furthermore, Chris lobbied the conference organizers to change the Gordon conference rules and I believe that after this meeting the Gordon conference was indeed more lenient on this topic. Chris leaves behind such a vast scientific and personal legacy and this story is just one small example that highlights his strong values and personal kindness.

**Fabrizio Chiti (Professor, University of Florence, Italy, as a PhD student in Oxford with Chris in 1996-1999 and then as a post-doc in Cambridge in 2002 for 9 months)**. I first met Chris in October 1996, when I started my Ph.D. with him in Oxford. It was really a wonderful time for me and three fantastic years. I remember enjoying any single discussion with him. Even though I was a young student without any experience, he let me follow my projects with absolute freedom, ready to correct my data misinterpretations or suggest (but never impose) promising and potentially interesting routes. I continued to collaborate with him until he died last September and I am proud to have published as many as 65 papers with him during these 23 years of activity, which is more, I gather, than any of his external collaborators. He had the power to make my results important, to extract a self-motivation from myself and to encourage my activity so that it has eventually provided more than it would have otherwise done. He was enthusiastic with my very few potentialities and blind to my many defects.

I learnt many things from Chris, including how to extract the most interesting results from a set of data, how to write a paper so that they could emerge from the whole body of experiments, how to motivate and gratify people in my lab and collaborators (albeit with <10% of his success, I should say), the importance of avoiding mentioning faults of other people in their absence, how to present results in conferences, how to write reference letters and many other things that these few lines cannot tell.

All these considerations can be extended, I am sure, to many other scientists who had the pleasure to be supervised by him. In fact, on top of his scientific success, a key descriptor of Chris' legacy is the large number of students and post-docs he followed, motivated, supported, encouraged, helped to gain self-confidence until, and after, they started their own scientific careers, independently of their personality, history and nationality. More than 100 former Ph.D./post-docs are presently covering group leader positions all over the world, and a few of them gained an international reputation in their fields of competence. People that were closest to Chris, know very well that this was his most profound pride. It is not surprising that his last thoughts before dying… have been for other people…

**Alfonso De Simone [Professor, Imperial College London, UK, in Chris' group in Cambridge in 2007-2014 supported by EMBO (2007-2009), Marie Curie (2009-2010), and EPSRC (2011-2014)]**. Chris has been a great inspiration for my entire career. I was inspired by his work before joining his group in 2007 and I have been even more inspired throughout the last 12 years that I worked closely with him. Working with Chris was extremely rewarding and has been transformative for me on both scientific and personal levels. He had a unique gift to see beyond the state of the art and pose the challenges for decades ahead in a way that no one else could do. Chris was a terrific mentor and motivator. I would have achieved a lot less in my career if it hadn't been for his constant support and drive to overcome our limits. I also witnessed the impact that Chris has had on the lives of hundreds of students and postdocs, and this is a beautiful legacy that will last a very long time.

**Tuomas P. J. Knowles (Professor, University of Cambridge UK, first as a postdoc and then as a colleague in 2004-2019)**. I first met Chris shortly after arriving in Cambridge in 2004. At that time, I was based in the Departments of Engineering and Physics for my Ph.D. work, but the interactions with Chris became rapidly scientifically and personally very important and inspired me to explore the applications of physical methods to chemical and biological problems.

Chris has been an inspiring model for so many of us on how to conduct interdisciplinary research and help students and post-docs to work together to tackle challenging problems. Chris was keen to talk to people independently of their seniority and always had time to help whenever problems arose. His attention to detail, both scientifically, but in writing papers in particular was renowned, and something that always impressed new students but also helped people structure their thoughts and generate excellent papers. He also had a way of combining applied research, especially in later years in the context of drug development for neurodegenerative diseases with his passion for fundamental chemistry in a way which was inspiring and I think a model of how translational research can be hugely successful.

I think Chris' legacy will live on through the work of the many students and post-docs whom he has mentored over the years, and of whose successes he was in a characteristically generous manner very proud. He has also transformed protein science, both in Oxford, Cambridge and worldwide by seeding original ideas, generating new ways of thinking of problems and inspiring the next generation of scientists.

**Janet R. Kumita (Principal Research Associate, University of Cambridge, UK, member of the Dobson group in 2003-2019)**. I first met Chris in early summer 2002, visiting Cambridge to discuss a postdoctoral fellowship to work in his lab. When I arrived, his meetings were running late and his diary was full of appointments, yet we sat for quite some time talking about science, my PhD research and UK/Canadian comparisons. He then stopped abruptly, asked if I wanted to go to lunch and whisked me through the streets of Cambridge to the college dining room (before lunch service ended). That day I felt honored to have had such a wonderful meeting—I hadn't realized that every subsequent meeting since that day would be just as personal, enlightening and positive. The list of all the things that Chris taught me is much too long, but one important thing I have learned is to be patient, understanding, optimistic and kind, in both my scientific and personal endeavors. Of course, Chris' biggest legacy is undoubtedly his scientific achievements, but perhaps equally important is how all of us, who were lucky enough to have been mentored by him, will continue our research and our lives following his impeccable example.

**Sheena E. Radford (Professor, Leeds, UK, worked with Chris in Oxford 1987-1995 as a postdoc and a Royal Society University Research Fellow)**. I first met Chris in 1987 when I went to work with him in the Inorganic Chemistry Laboratory at the University of Oxford. In those days, protein work was a small part of Chris' lab, with just a handful of us working on lysozyme and alpha-lactalbumin, whilst others worked on the polymerization of inorganic materials. We had very little in the lab, a pipette, distilled water, and lysozyme bought by the gram from Sigma. But we had such a great time, learning how to fold proteins, developing hydrogen exchange to capture folding intermediates, using NMR and later, when Carol Robinson joined the group, using mass spectrometry. Folding lysozyme from various birds from Lady Amherst Pheasant to Emu (as we couldn't do site directed mutagenesis of lysozyme in those days). The discovery that human lysozyme is involved in amyloid disease was perhaps the most major breakthrough, and the rest, as you will all know, is history. But it showed that all those lysozyme folding experiments led to new science and a new field for us, that we could never have predicted or even imagined at the time.

I learned so much it cannot be written within a small space! But, scientifically, he taught us to always see the bigger picture, to put our results in a global context and to tell the final story well. He was a wonder with the pen! He also led by example—he was kind to all, ensuring we all reached our full potential in science and as human beings. He was also of the firm opinion that ones' best science is often the hardest to publish: that has kept me going throughout my own scientific career!

Well, apart from the >800 manuscripts we should all re-read, Chris has left generations of excellent scientists across the world working on the one of the most fascinating and biggest problems facing humankind—protein aggregation and amyloidosis. We are all followers of a great man, and it is up to us all now to continue in this field, working together globally as he would have wished us to do to crack this problem. And, if we can capture an ounce of Chris' wisdom, insightfulness and wonderful dry sense of humor that kept us all going in difficult times, on long journeys, and at many a conference dinner, we will have all done well and made him proud.

**Dame Carol V. Robinson (Professor, University of Oxford, UK, in Chris' group in 1991-2000)**. I first encountered Chris in 1992 after returning from my 8-year career break. I was seriously lacking in confidence at that time and I was working as a post-doctoral researcher providing a mass spectrometry service to the OCMS. I was extremely grateful for this opportunity to return to the lab but Chris thought I should aim higher. Within a few years he had persuaded me to apply for a Royal Society University Research Fellowship. With this in place, I launched my independent career very much under his guidance and secured a post in Cambridge when he moved. By the early 2000's our research interests started to diverge and we became colleagues in the Department of Chemistry at Cambridge for 9 years prior to my move back to Oxford. Over the last decade Chris remained a much-valued mentor to me. He taught me to focus on what I did know and not what I did not. For me Chris' legacy is to continue mentoring students and researchers in the way that he did for me.

**Xavier Salvatella (ICREA Research Professor, Institute for Research in Biomedicine and Barcelona Institute of Science and Technology, Spain, worked with Chris in 2003-2008)**. I first met with Chris in a small meeting on amyloids organized by Tom Mc Leish in London, probably in 2002, shortly after Chris had moved his lab to Cambridge. Although we had never met Chris had already accepted me as postdoc and I had obtained a fellowship to work with him. I still had not finished writing my thesis, though, and the first words that Chris said to me after I introduced myself after his talk were “So, when are you coming?” I eventually made it to Cambridge and ended up spending 5 years working in the unique environment that he created for ambitious young scientists such as myself, a period that shaped my attitude toward research, science and also life.

On reflecting on what I learned from Chris during these years I have come to realize that, in addition to science and the business of science, Chris taught me, often by example, the importance of resilience, perspective, hard work and kindness in the scientific enterprise; in fact Chris continues to teach me to this day, as I often recall discussions with him, or pieces of advice that he gave me, when facing challenges and taking decisions.

Chris's numerous and important contributions to science, aimed at gaining a detailed understanding of the mechanisms of protein folding, misfolding and aggregation into amyloid fibrils, are based on the idea that such knowledge is important by itself but also as a platform for the discovery of drugs to, in the future, manage some of the most challenging diseases that we face as a species such as Alzheimer's disease, a modern plague as he liked to call it. It is terribly sad that he will not be able to see the practical applications of his life's work but others will carry on, inspired by him, and those, like myself, who have worked with him will be very proud when they succeed.

**Karen Valelli** (**Chris' Personal Assistant, University of Cambridge, UK, with Chris 2007-2019**). My first meeting with Chris is too far back to remember. But I remember the first time we met at the Master's Lodge, St. John's College, very clearly. He had just taken up the position and I took him some coffee and cakes to eat during our first meeting there as a little celebration. I was in absolute wonderment at the beauty of the stately home he was now living in. We sat back in our chairs and I exclaimed, “Wow, this is AMAZING, Chris.” He sipped his coffee and replied, “Yes, it IS, isn't it!”, and we both laughed. We laughed a lot throughout all those years. What I learned from Chris was the power of telepathy. That's mostly how we conducted our business relationship during the past 12 years, given that he was always traveling. And it worked very successfully! His legacy is kindness and nurturing and all of us here aim to carry that on in our lives so that everyone feels well-supported.

**Michele Vendruscolo (Professor, University of Cambridge, UK, Chris' colleague 1999-2019)**. Chris was a great scientist because he had a deep and clear vision and the ability of building the structure to pursue it. But most importantly, his achievements went together with his unwavering determination to support his fellow researchers and empower them to fulfill their potential. He had the rare ability to meet people and fully listen to them for hours, often with transformational effects on their lives. He has been an inspiring person whose spirit will remain with us for the rest of our lives.

**Annalisa Pastore (Professor, King's College London, UK, never in Chris' lab but adopted by them when postdoc in Iain D. Campbell in Oxford in 1985-1987)**. I do not recall my first meeting with Chris but it certainly was in 1986 when I was a post-doc in Iain Campbell's lab. We were next door to the NMR instruments and would meet all users. I therefore knew most of the people from the Campbell's, Dobson's, and Bob Williams' laboratories, the three major groups working in NMR at the time. People would joke about Chris: what do I do? I work in Chris Dobson's group and work on lysozyme, what else? I then became the local “expert” of molecular dynamics calculations. These were early days for the use of this technique in structural calculations. Through this role I would assist also people from other groups and was adopted by several them as part of a larger group without boundaries. At some point I turned to Chris for a problem or an advice. He was very supportive and remained so in several more difficult steps of my career. Supportive and constructive: these are the two words that I would associate with him.

There is a very strong lesson I learned from Chris: to always look for new techniques, never use only one. I remember the first article on lysozyme using mass spectrometry. For me used to people who would work for the technique and not for the problem, this was a sudden revelation. There is nothing wrong with working with the same technique for the whole life and try to push its potentialities to new limits. But for me what Chris was doing was far more congenial: he was always introducing yet another tool to his studies with an incredible degree of creativity. Later on he presented the beautiful work on fly models of degeneration: I distinctively remember when Chris showed flasks full of Drosophila flies at a meeting able to move more or less quickly according to the drug they had been treated. I remember thinking “Only Chris could do this!” I tried, in my work, to do the same and look always for something new.

I frankly think that, had he lived longer, Chris would have got the Nobel prize. He certainly deserved it. His work has been, is and will remain inspirational for generations of researchers all over the world. His contributions to Science go far beyond protein folding and amyloids. Not only he created new interests but also he introduced a different way to introduce a particular attention to the role of physics and computational biology in wet biology. This way of thinking will hopefully inspire more people in the future.

**Gian Gaetano Tartaglia (ICREA professor at Centre for Genomic Regulation, Barcelona Institute of Science and Technology Spain and Rome Sapienza Professor, Italy, in Chris' group 2005-2010)**. The first encounter I had with Chris was in 2004. At that time, I was in Zurich working on the experimental characterization of artificial peptides designed to have a specific fold. We discovered that all the peptides were aggregating in the very way that Chris had described in his papers with Fabrizio. Thanks to this simple and great intuition, my results started to make sense and I began my career. In 2005, I moved to Cambridge to work together with Chris and Michele. Chris knew how to get to the point and gratify the interlocutor by posing the right questions in the middle of the discussion. His cleverness and great intuition could go beyond what others would see. His “generic hypothesis” that each protein can adopt the amyloid state has so many implications for Physics, Chemistry, and Biology.

Chris trained hundreds of scientists. His legacy lives in us and all the new researchers that work in the many avenues that he opened up.

## Author Contributions

All authors listed have made a substantial, direct and intellectual contribution to the work, and approved it for publication.

### Conflict of Interest

The authors declare that the research was conducted in the absence of any commercial or financial relationships that could be construed as a potential conflict of interest.

